# Dietary supplementation of *Macleaya cordata* extract and *Bacillus* in combination improve laying performance by regulating reproductive hormones, intestinal microbiota and barrier function of laying hens

**DOI:** 10.1186/s40104-022-00766-4

**Published:** 2022-10-13

**Authors:** Fei Wang, Peng Zou, Shujie Xu, Qi Wang, Yuanhao Zhou, Xiang Li, Li Tang, Baikui Wang, Qian Jin, Dongyou Yu, Weifen Li

**Affiliations:** 1 Key Laboratory of Animal Molecular Nutrition of Education of Ministry, National Engineering Laboratory of Biological Feed Safety and Pollution Prevention and Control, Key Laboratory of Animal Feed and Nutrition of Zhejiang Province, Institute of Animal Nutrition and Feed Sciences, College of Animal Sciences, Zhejiang University, Hangzhou, 310058 China; 2 Hainan Institute, Zhejiang University, Yazhou Bay Sci-Tech City, Yongyou Industry Park, Sanya, 572000 China

**Keywords:** *Bacillus*, Intestinal microbiota, Laying hens, Laying performance, *Macleaya cordata* extract, Reproductive hormones

## Abstract

**Background:**

This study aimed to investigate whether the combination of *Macleaya cordata* extract (MCE) and *Bacillus* could improve the laying performance and health of laying hens better.

**Methods:**

A total of 360 29-week-old Jingbai laying hens were randomly divided into 4 treatments: control group (basal diet), MCE group (basal diet + MCE), Probiotics *Bacillus* Compound (PBC) group (basal diet + compound *Bacillus*), MCE + PBC group (basal diet + MCE + compound *Bacillus*). The feeding experiment lasted for 42 d.

**Results:**

The results showed that the laying rate and the average daily egg mass in the MCE + PBC group were significantly higher than those in the control group (*P* < 0.05) and better than the MCE and PBC group. Combination of MCE and *Bacillus* significantly increased the content of follicle-stimulating hormone (FSH) in the serum and up-regulated the expression of related hormone receptor gene (estrogen receptor*-*β*, FSHR* and luteinizing hormone/choriogonadotropin receptor) in the ovary of laying hens (*P* < 0.05). In the MCE + PBC group, the mRNA expressions of zonula occluden-1, *Occludin* and *mucin-2* in jejunum was increased and the intestinal epithelial barrier detected by transmission electron microscopy was enhanced compared with the control group (*P* < 0.05). In addition, compared with the control group, combination of MCE and *Bacillus* significantly increased the total antioxidant capacity and catalase activity (*P* < 0.05), and down-regulated the mRNA expressions of inflammation-related genes (interleukin-1β and tumor necrosis factor-α) as well as apoptosis-related genes (*Caspase 3*, *Caspase 8* and *P53*) (*P* < 0.05). The concentration of acetic acid and butyric acid in the cecum content of laying hens in the MCE + PBC group was significantly increased compared with the control group (*P* < 0.05).

**Conclusions:**

Collectively, dietary supplementation of 600 μg/kg MCE and 5 × 10^8^ CFU/kg compound *Bacillus* can improve laying performance by improving microbiota to enhance antioxidant capacity and intestinal barrier, regulate reproductive hormones and the concentration of cecal short-chain fatty acids of laying hens, and the combined effect of MCE and *Bacillus* is better than that of single supplementation.

**Supplementary Information:**

The online version contains supplementary material available at 10.1186/s40104-022-00766-4.

## Background

The plant extracts [1], probiotics [2], acidifiers [3], antimicrobial peptides [4, 5] and other dietary additives have been widespread in husbandry to control infection of pathogens and improve growth performance of animals in recent years in China.

*Macleaya cordata* is a crucial perennial herbaceous medicinal plant and belongs to the genus of *Macleaya* (Papaveraceae). *Macleaya cordata* extract (MCE) contains a variety of natural bioactive alkaloids, mainly including sanguinarine (SG), chelerythrine (CHE) and allocryptopine [6], which have antimicrobial [7], anti-inflammatory [8, 9], antioxidant [10] and immune regulation [11] properties. It has been reported that MCE was well tolerated when feeding poultry [12], pigs [13], beef cattle [14] and dairy cows [15], and played an important role in promoting livestock and poultry production. For example, MCE can significantly reduce diarrhea score and enhance intestinal barrier function of piglets [16]. MCE can improve the growth performance of chickens by increasing the abundance of intestinal lactobacillus and the biosynthesis pathways of amino acids, vitamins and secondary bile acids of microbiota [17]. Moreover, MCE can reduce enterotoxigenic *Escherichia coli* (ETEC) induced oxidative stress by reducing the abundance of methane dicarboxylic acid and enhancing the activities of catalase and glutathione peroxidase [10]. In addition, the characteristics of plant-derived products are natural, multi-functional and low in toxicity [18]. Our previous studies have shown that MCE has positive effects on performance and body health of laying hens (unpublished data).

Probiotics have the functions of maintaining the balance of intestinal microbiota, inhibiting the colonization of pathogenic bacteria, improving the intestinal mucosal structure, promoting the digestion and absorption of nutrients, enhancing the immune function of the body and preventing the death of animals [19–21]. *Bacillus* is preferred as a feed supplement due to its higher resistance to harsh environment [22]. Both *Bacillus amyloliquefaciens* SC06 (BaSC06) and *Bacillus subtilis* 10 (B10) belong to the genus of *Bacillus*, a Gram-positive bacterium, which is commonly used in animal feed products [23]. *B. amyloliquefaciens* and *B. subtilis* are similar in morphology, culture characteristics as well as physiological and biochemical characteristics, and can produce a variety of extracellular enzymes, such as α-amylase, β-amylase, cellulase and protease, which can enhance intestinal digestibility, nutrient absorption and immune function [24–26]. The results of our previous studies showed that BaSC06 can improve growth performance of finishing pigs by increasing antioxidant capacity, digestion and absorption of nutrients and intestinal barrier function [27]. In addition, BaSC06 can improve growth performance and meat quality of broilers by increasing intestinal barrier and immunity [28]. B10 can significantly improve the growth performance and intestinal morphology of broilers by regulating the intestinal microbiota [29].

It was reported that dietary mixtures of plant extracts and probiotics had significant effects on the performance and immune function of laying hens [30–33]. However, less information has been reported on how the combination of MCE and *Bacillus* improves the performance of laying hens. Here, we hypothesized that the combination of MCE and *Bacillus* can significantly improve laying performance, and the combined effect of MCE and *Bacillus* is better than that of single supplementation. To test the hypothesis, we carry out the present study.

## Materials and methods

All experimental procedures were conducted in accordance with the Animal Welfare Committee guidelines and the experimental protocol was approved by the Animal Care and Use Committee of Zhejiang University (Hangzhou, China).

### MCE and probiotic preparation

MCE preparation consists of 1.2 g MCE and 998.8 g starch. Probiotics *Bacillus* Compound (PBC) preparation (including BaSC06 and B10, both isolated and preserved by our laboratory) was prepared and provided by our laboratory. The BaSC06 and B10 were separately cultured in Luria–Bertani (LB) broth at 37 °C overnight under aerobic conditions. The BaSC06 and B10 pellets were collected after centrifugation at 3500 × *g* for 10 min at 4 °C, and then washed three times with sterile phosphate-buffered saline (PBS, pH 7.2). Finally, the concentration was constantly checked by the spreading plate method. Then BaSC06 and B10 pellets were diluted by starch to make PBC preparation with about 1 × 10^9^ colony-forming units (CFU)/g, respectively. Finally, the two *Bacillus* preparation were mixed in a ratio of 1:1 to form compound probiotics preparation contained 1 × 10^9^ CFU/g compound *Bacillus*. MCE + PBC preparation was added with 1.2 g MCE on the basis of Probiotics *Bacillus* Compound.

### Experimental design

In this experiment, a total of 360 29-week-old commercial laying hens of Jingbai strain with the similar performance were randomly allotted to four dietary treatment groups, comprising the control and three experimental groups. Each of the groups consisted of 6 replicates and each replicate contained 15 laying hens. The control group was fed a basal diet. The MCE group fed a basal diet supplemented with 600 μg/kg MCE. The PBC group was fed a basal diet supplemented with 5 × 10^8^ CFU/kg compound *Bacillus*. The MCE + PBC group was fed a basal diet supplemented with 600 μg/kg MCE and 5 × 10^8^ CFU/kg compound *Bacillus*. The PBC and MCE were first mixed with the premixes and then with the other ingredients to make feed mixing uniformity higher. The basic corn-soybean meal diets were formulated to meet or exceed the nutritional requirements for laying hens calculated according to The National Research Council (NRC, 1994) recommended (Table 1) [34].

**Table 1:** Ingredients and nutrient contents of the basal diet (as-fed basis)

Items	Value
Ingredients, %	
Corn	57.00
Soybean meal (46% CP)	24.00
Wheat middling	5.50
Soybean oil	1.00
Limestone	9.00
Dicalcium phosphate	1.00
Salt	0.30
*DL*-methionine	0.12
Lysine-HCl	0.08
Premix^a^	2.00
Total	100.00
Nutrient level^b^	
Metabolizable energy, Mcal/kg	2.70
Crude protein, %	16.43
Lysine, %	0.89
Methionine, %	0.40
Cysteine + methionine, %	0.75
Calcium, %	3.62
Total phosphorus, %	0.56
Available phosphorus, %	0.35

^a^The premix provided the following per kg of the diet: iron, 60 mg; copper, 10 mg; manganese, 80 mg; zinc 80 mg; iodine 0.3 mg; vitamin A, 12,500 IU; vitamin D_3_, 4000 IU; vitamin K_3_, 2 mg; thiamine, 1 mg; riboflavin, 8.5 mg; calcium pantothenate, 50 mg; niacin acid, 32.5 mg; pyridoxine, 8 mg; folic acid, 5 mg; B_12_, 5 mg; choline chloride, 500 mg; phytase, 1000 IU

^b^The nutrient levels were calculated values

Used staggered three-layer cages, each independent cage put 5 laying hens, one week before entering the chickens, the cage was disinfected according to the usual procedures. Sixteen hours of light a day, natural light supplemented by artificial light. The feeding experiment lasted for six weeks. The first week was considered an adaptation period, and the next five weeks were a formal experiment. During the preliminary experiment, we observed the laying rate of laying hens and adjusted each group so that there was no statistical difference in laying rate between groups. During the entire experiment period, they were free to eat and drink, and were fed twice a day at 7:30 in the morning and 15:00 in the afternoon. Immunization was carried out according to the routine immunization program.

### Sample collections

At the end of the experiment (42 d), all laying hens were deprived of feed for 12 h, but water was offered ad libitum. Then six laying hens (one hen per replicate) were selected and marked from each treatment group randomly, weighed and blood samples were collected before slaughter from the wing vein using 5-mL vacuum blood tubes. After the serum was separated naturally, it was centrifuged for 10 min (3000 × *g*) to separate out the serum. Pure serum samples were aspirated by pipette, stored in 1.5-mL eppendorf tubes at –80 °C. In addition, laying hens were euthanized to enable the collection of tissues. The jejunum was ligated and separated, the middle part of the intestine segment was taken (the head and tail of each segment were cut off 1 cm each), and approximately 1 mm long near the posterior end of the intestine segment was divided into two parts. One segment was fixed in 4% paraformaldehyde for hematoxylin and eosin (H&E) staining, the other segment was fixed in 2.5% buffered glutaraldehyde for transmission electron microscopy (TEM). The rest of the jejunum segment and the whole caecum as well as the intact ovary of laying hens was sampled, snapped frozen in liquid nitrogen and then stored at –80 °C for further analysis.

### Production performance assay

Egg production and mass were recorded daily (at 8:00), and feed consumption was recorded weekly on a replicate basis (6 replicates per dietary treatment) to calculate the laying rate, average daily egg mass, average daily feed intake, and feed conversion ratio (feed/egg: g/g) as follows: laying rate (LR) (%) = Total number of eggs/laying hens number/days(d) × 100; feed conversion ratio (FCR) = Total feed consumption (g)/total egg weight (g); average daily egg mass (ADEM) (g/hen/d) = Total egg mass (g)/laying hens number/days (d); average daily feed intake (ADFI) (g/hen/d) = (total final feed intake (g)—total initial feed intake (g))/days (d)/laying hens number.

### Serum biochemical parameters

The activities of lactic dehydrogenase (LDH), myeloperoxidase (MPO), alanine aminotransferase(ALT), aspartate aminotransferase(AST), alkaline phosphatase (AKP), total antioxidant capacity (T-AOC), total superoxide dismutase (T-SOD), catalase (CAT) and glutathione peroxidase (GSH-Px) as well as the concentration of total cholesterol (TC), total protein (TP), albumin(ALB), blood urea nitrogen (BUN), uric acid (UA) and malondialdehyde (MDA) in the serum by using commercial kits based on manufacturer’s guidelines (Jiancheng Bioengineering Institute, Nanjing, Jiangsu, China), which use colorimetric methods and measured with a spectrophotometer. All experiments had six replicates.

### ELISA Assay

The concentrations of follicle-stimulating hormone (FSH, H101-1–2) and luteinizing hormone (LH, H206-1–2) were determined by using an enzyme-linked immunosorbent (ELISA) kits (Jiancheng Bioengineering Institute, Nanjing, Jiangsu, China) according to the manufacturer’s instruction. Briefly, serum samples were added into enzyme wells, which has been pre-coated with antibodies specific for FSH and LH, then added recognition antigen labeled by horse radish peroxidase (HRP); after been incubated 30 min at 37 °C, both compete with solid phase antigen and formed immune complex; after been washing by phosphate buffered saline tween (PBST), the combined HRP catalyzes Tetramethy1 benzidine (TMB) into blue, and turns into yellow by the action of acid; it has absorption peak under 450 nm wavelength, and the absorbance of each well was determined using a SpectraMax M5 (Molecular Devices, San Jose,CA, USA). All experiments had six replicates.

### Hematoxylin and eosin (H&E) staining

The H&E staining was performed as previously described, with minor modifications [35]. Jejunum samples of laying hens were fixed with 4% paraformaldehyde, embedded in paraffin, sliced, dehydrated and stained with hematoxylin and eosin. The image was taken using Olympus Microsystem (Tokyo, Japan).

### Transmission electron microscopy (TEM)

After fixation in 2.5% glutaraldehyde buffer, jejunum tissue was washed 3 times every 15 min in 0.1 mol/L cold phosphate buffer. The tissue was fixed in 0.1% osmium tetroxide (OsO_4_) cold buffer for 2 h, and then washed with phosphate buffer. After rapid dehydration in successively increasing ethanol solutions (30%, 50%, 70%, 95%, and 100%), the tissues were transferred to a 1:1 mixture of epoxy propane and epoxy aldehyde resin. After embedding, ultrathin Sects. (60–100 nm) were cut with an LKB Nova ultra-slicer (Leica Microsystems, Buffalo Grove, IL, USA) and stained with uranyl acetate. Electron microscopic images of intestinal mucosal cells and microvilli were taken by transmission electron microscope (JEOL, Tokyo, Japan) at 80 kV.

### RNA Extraction and RT-qPCR

The RNA extraction was performed using RNAiso plus (Takara, Dalian, Liaoning, China) according to manufacturer's protocols, then utilize the PrimeScript II 1^st^ Strand cDNA Synthesis Kit (Vazyme, Nanjing, Jiangsu, China) to synthesize cDNA based on the manufacturer’s manual. The RT-qPCR analysis was conducted using HiScript II One Step RT-qPCR SYBR Green Kit (Vazyme, Nanjing, Jiangsu, China) based on the manufacturer’s manual by the StepOne Plus Real-Time PCR system (Applied Biosystems, Carlsbad, CA, USA). The primers used in this study were designed using the NCBI Primer-Blast tool. All primer sequences for target genes are listed in Supplementary Table S1. The fold changes were calculated after normalizing to the housekeeping gene β-actin, and the 2^−ΔΔCt^ method was used to estimate mRNA abundance [36]. All experiments were performed in triplicate.

### Microbial analysis

Microbial genomic DNA was extracted under sterile conditions from the cecal content of laying hens using the TIANamp Stool DNA Kit (Tiangen, Beijing, China) according to the manufacturer’s instructions. The quality of extracted DNA was checked by agarose gel electrophoresis and spectrophotometric analysis. The V3–V4 region of the 16S rRNA gene was amplified using the primer pair 341F/805R, and sequencing was performed on MiSeq platform (Illumina Inc., San Diego, CA, USA). Sequences were filtered and clustered into operational taxonomic unit (OTU) with 97% similarity by QIIME software (version 1.9.1).

Alpha diversity (Ace, Chao1, Shannon and Simpson) was calculated to reflect bacterial diversity and richness. Principal coordinate analysis (PCoA) which is based on Bray-Curtis was performed to get principal coordinates and visualized from complex data. The relative abundance of microbiota was examined at different taxonomic levels. The histogram of linear discriminant analysis (LDA) distribution was implemented using LDA effect size analysis (LEfSe) software. The relative abundance of significant differences in phylum, class, order, and OTU levels was calculated by the one-way analysis of variance (ANOVA). The 16S rRNA gene sequencing information was analyzed by PICRUSt2 to predict biological functions and metabolic pathways (KEGG database) of the bacterial community of intestinal contents samples of laying hens.

### Analysis of SCFAs in the cecal contents by gas chromatography

The protocol for analysis of SCFAs in the cecal contents by gas chromatography was conducted according to previous study described [37, 38]. Briefly, 100 mg of cecal content was homogenized with 1 mL of sterile PBS, centrifuged at 12,000 r/min and 4 ℃ for 10 min. Then, 500 μL aliquot of the supernatant fluid was diluted with 100 μL of 25% (w/v) metaphosphoric acid solution. The mixture was incubated at –20 ℃ for 24 h, then centrifuged at 4 ℃ and 12,000 r/min for 10 min. Finally, the supernatant was filtered through a 0.22-μm syringe filter and injected into Shimadu GC-2030 ATF instrument for SCFAs detection. The carrier gas was N_2_ (pressure, 12.5 Mpa and flow, 18 mL/min), the temperature of the injector and detector was 180 ℃, and the column was gradually heated from 80 ℃ to 170 ℃ at a rate of 4 ℃/min.

### Statistical analysis

All data was analyzed by SPSS 25.0 software (SPSS Inc., Chicago, IL, USA) to detect a significant difference with one-way analysis of variance (ANOVA) followed by LSD and Tukey post-hoc tests. Results were expressed as means ± standard error of mean (SEM), and the values of *P* < 0.05 was considered to indicate a statistically significant difference. Graphs were generated by GraphPad Prism 8.0 software (GraphPad Software, San Diego, CA, USA).

## Results

### Laying performance

Dietary supplementation of MCE and PBC complex significantly increased LR and ADEM compared with the control group (*P* < 0.05). Compared with PBC group, LR in the MCE + PBC group was significantly increased (*P* < 0.05). But, ADFI and FCR of laying hens fed with MCE and PBC separately or in combination were not statistically (*P* > 0.05) different from the control group (Table 2).

**Table 2:** Laying performance of laying hens^1^

Item	Control	MCE	PBC	MCE + PBC	SEM	*P*-value
LR, %	91.52^b^	92.70^ab^	92.00^b^	94.48^a^	0.01	0.003
ADEM, g/hen/d	52.28^b^	52.86^ab^	52.56^ab^	53.73^a^	0.38	0.026
ADFI, g/hen/d	111.42	111.18	111.63	112.43	0.59	0.503
FCR, g^feed^/g^egg^	2.13	2.10	2.12	2.09	0.02	0.320

^1^Results are the mean of 6 replicates of 15 laying hens each. *LR* laying rate, *ADEM* average daily egg mass, *ADFI* average daily feed intake, *FCR* feed conversion ratio *SEM* standard error of mean

^a^^−^^b^Value differences in the same row differ significantly (*P* < 0.05)

**Table 3:** Serum hormone levels of laying hens^1^

Item	Control	MCE	PBC	MCE + PBC	SEM	*P*-value
FSH, mIU/mL	18.12^b^	22.32^ab^	21.82^ab^	24.01^a^	1.50	0.028
LH, mIU/mL	13.99	15.23	14.78	17.22	1.20	0.281

^1^Results are the means of each group of 6 laying hens. *FSH* follicle-stimulating hormone, *LH* luteinizing hormone, *SEM* standard error of mean

^a^^−^^b^Value differences in the same row differ significantly (*P* < 0.05)

### Hormone indices

**Fig. 1:**
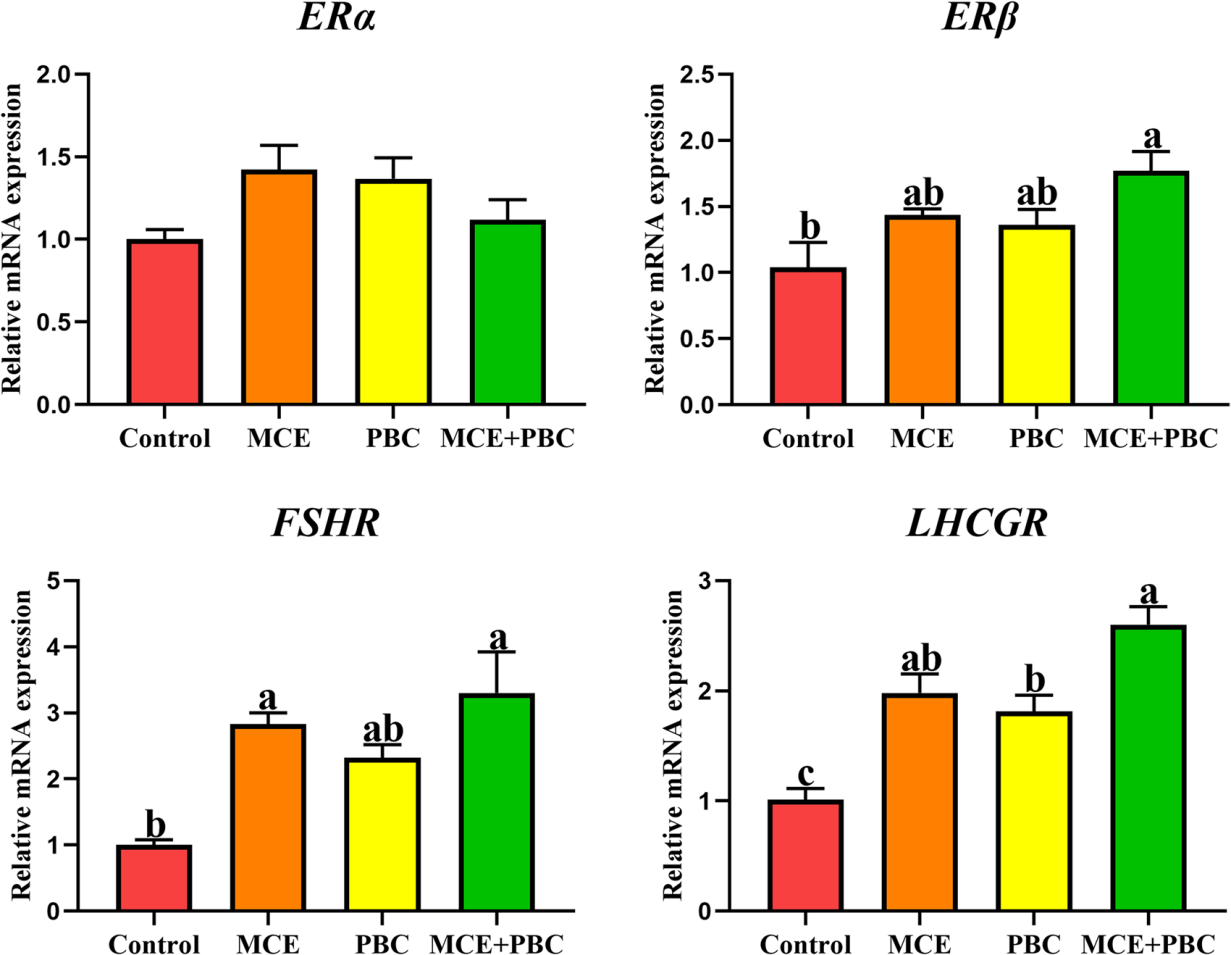
The relative mRNA expressions of *ER-α*, *ER-β*, *FSHR* and *LHCGR* were analyzed by real-time qPCR. Data are presented as means ± SEM (*n* = 3). Different lowercase letters indicate a significant difference (*P* < 0.05). *ER*: estrogen receptor; *FSHR*: follicle-stimulating hormone receptor; *LHCGR*: luteinizing hormone/choriogonadotropin receptor

FSH and LH are the main hormones that affect egg production. The content of FSH and LH in serum detected by ELISA kits showed that FSH in the MCE + PBC group was significantly increased compared with the control group (*P* < 0.05), but there was no significant difference in LH among all groups (*P* > 0.05) (Table 3). Then we detected the mRNA expression of related hormone receptor on ovary, and found that compared with the control group, the MCE + PBC significantly increased the mRNA expression of *ER-β* (*P* < 0.05), the MCE and the MCE + PBC both increased the mRNA expression of *FSHR* (*P* < 0.05), and all treatment increased the mRNA expression of *LHCGR* (*P* < 0.05), but had no significant effect on *ER-α* (*P* > 0.05) (Fig. 1).

### Intestinal microbiota

**Fig. 2:**
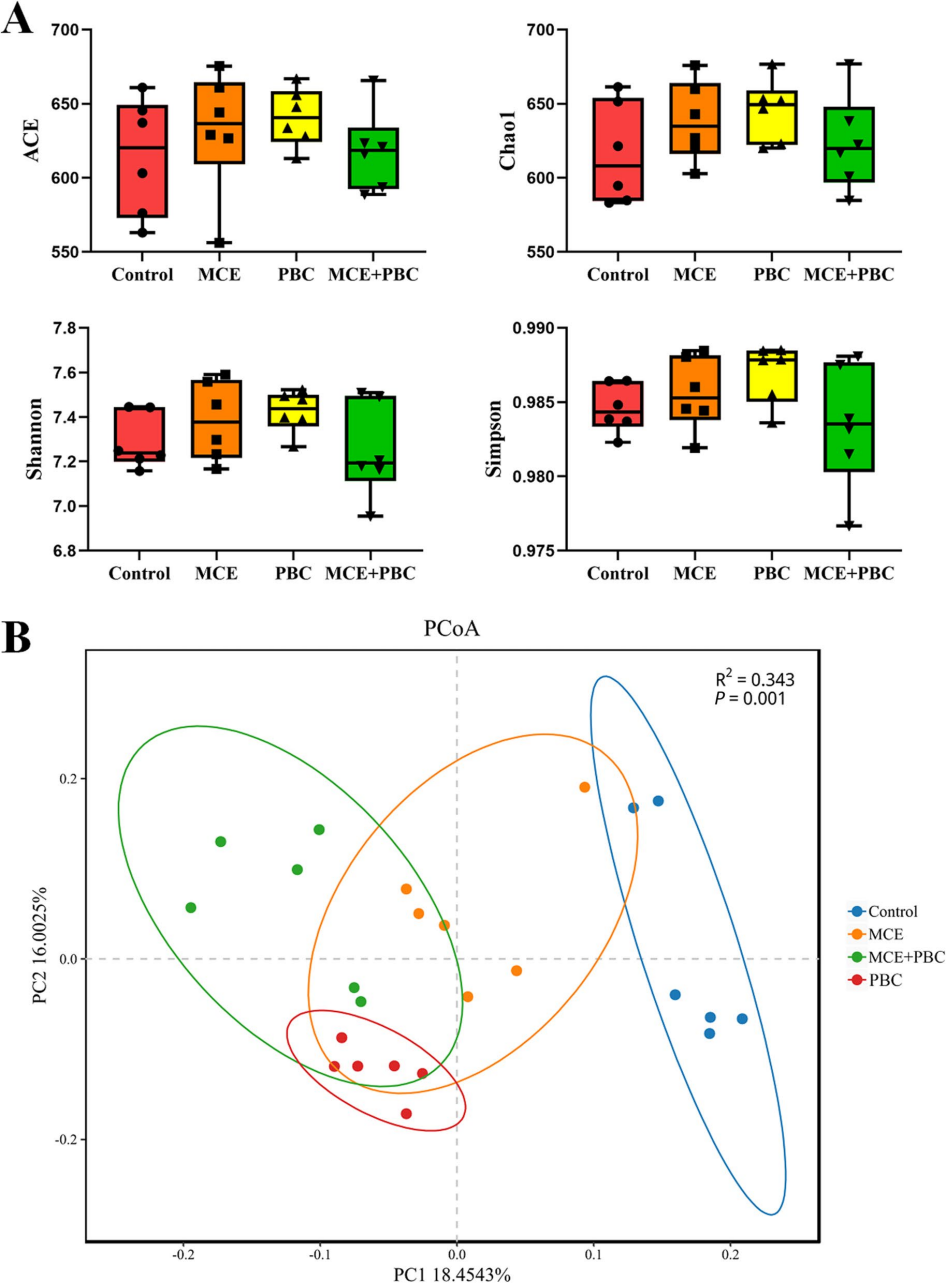
Diversity analyses of microbial communities among groups. (**A**) Alpha diversity (Ace, Chao1, Shannon and Simpson) (**B**) Principal coordinates analysis (PCoA) of microbial communities among groups based on Bray–Curtis distance. (*n* = 6 per group)

In this study, all treatments had no effect on the alpha diversities of the intestinal microbiota in laying hens (*P* > 0.05) (Fig. 2A). PCoA of microbial community based on Bray–Curtis distance showed that microbial community structure had changed with treatment, which are divided into two distinct types. The microbial communities of laying hens in control formed a cluster and formed another cluster in the MCE, PBC and MCE + PBC groups (Fig. 2B).

**Fig. 3:**
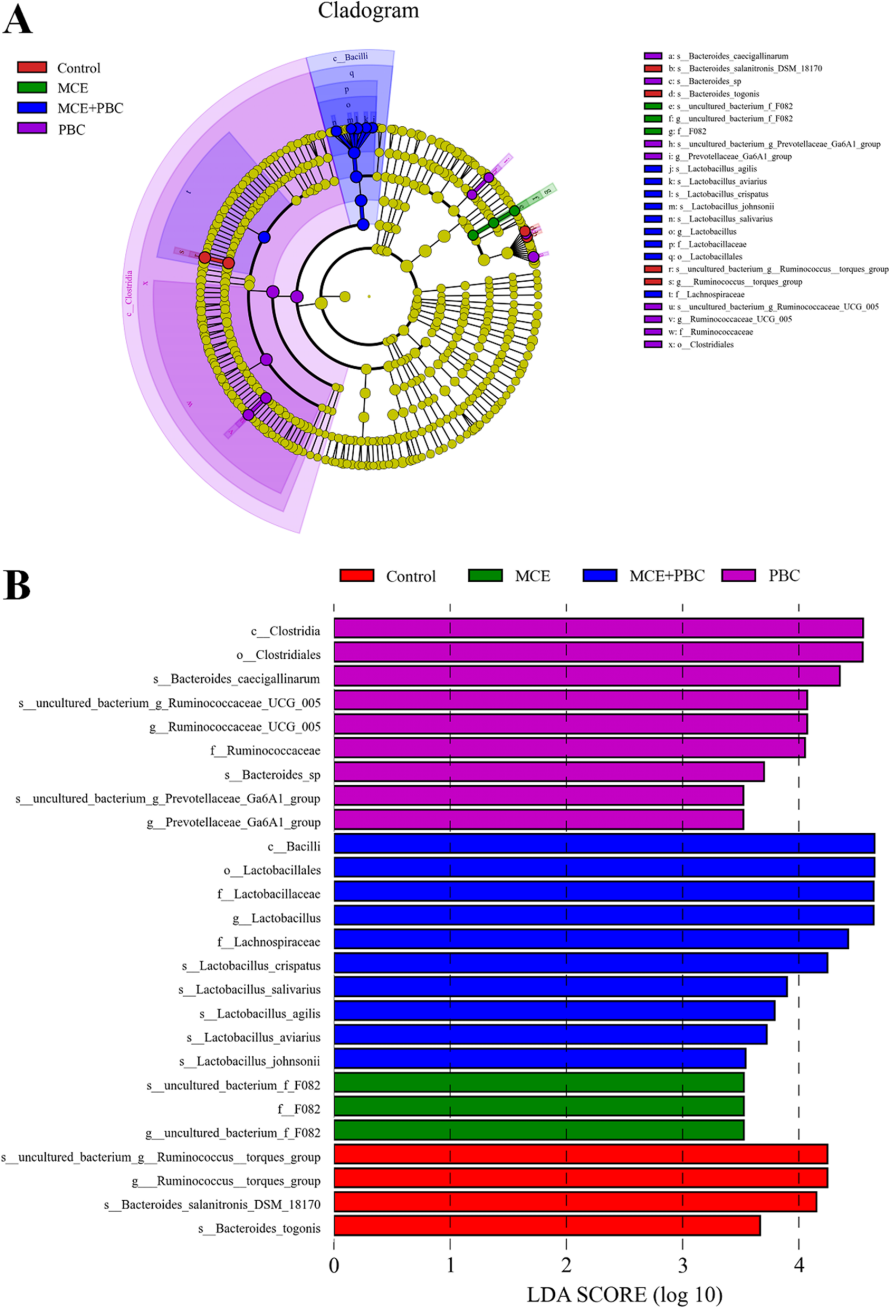
Linear discriminant analysis (LDA) effect size (LEfSe) analysis of the cecum microbial community in the four groups. (**A**) The cladogram of LEfSe analysis. (**B**) The histogram of LEfSe analysis. p_: phylum level; c_: class level; o_: order level; f_: family level; g_: genus level; s_: species

LEfSe analysis further found that there were significant differences in the relative abundance of bacteria in the cecal microbiota among the four groups. Bacterial taxa with LDA score greater than 3.5 are selected as biomarker taxa, and we found that 26 taxa biomarkers in the four groups (Fig. 3), which mainly belong to the class of Clostridia and Bacilli, the order of Clostridiales and Lactobacillales, and the family of Ruminococcaceae, Lactobacillaceae, Lachnospiraceae and F082.

**Fig. 4:**
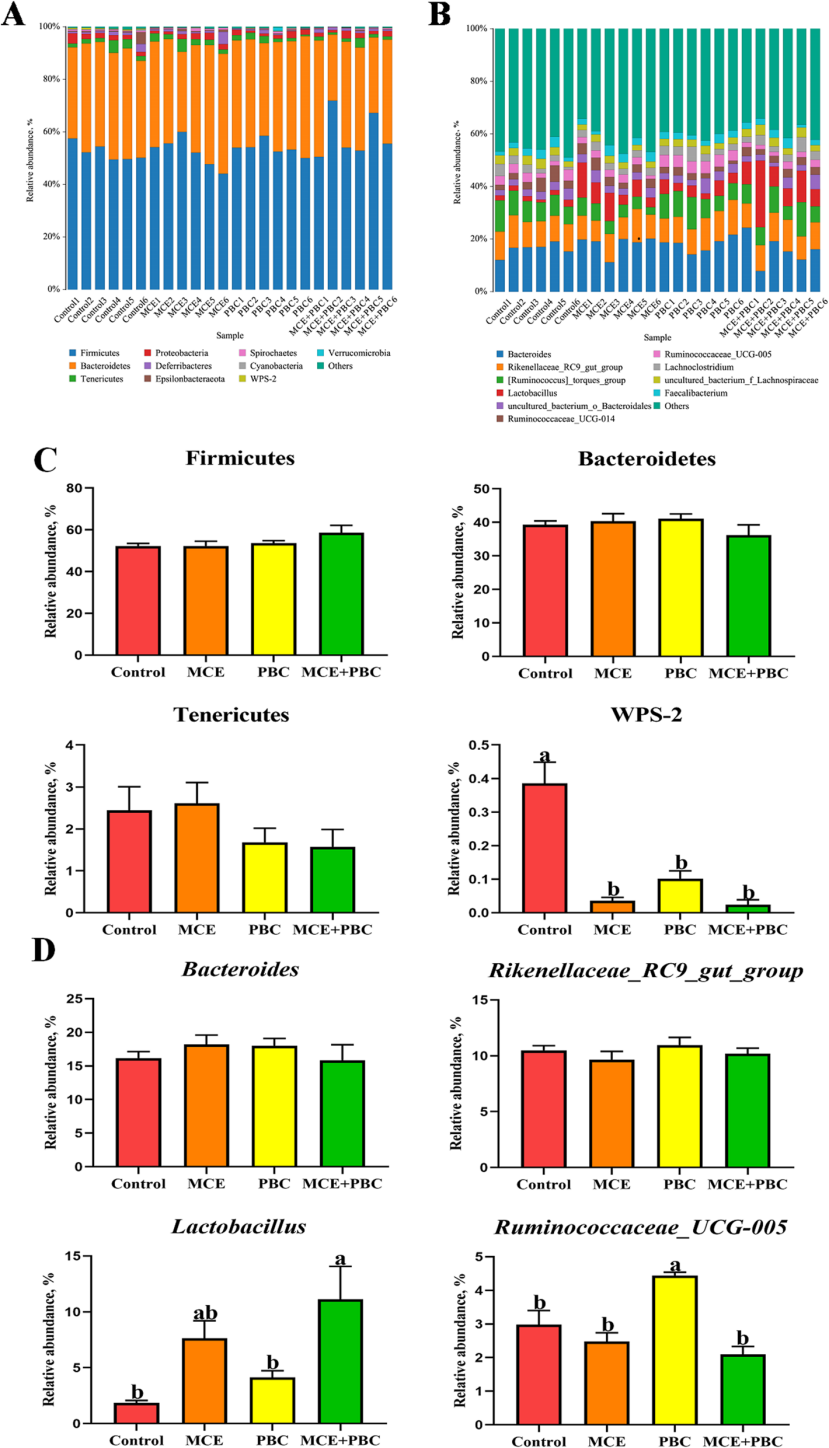
(**A**–**B**) Average relative abundance of microbial species in the cecum at the phylum level (**A**) and genus level (**B**). (**C**–**D**) Relative abundance of microbial communities in the cecum contents at the phylum level (**C**) and genus level (**D**). Different lowercase letters indicate a significant difference (*P* < 0.05)

The relative abundances of different phyla and genus were presented in Fig. 4A-B. Differential analysis results further showed that, compared with the control group, at the phylum level there were no significant differences in the relative abundance of Firmicutes, Bacteroidetes and Tenericutes among all treatment groups, but reduced the relative abundance of WPS-2 significantly (Fig. 4C). At the genus level, the MCE + PBC group significantly increased the relative abundance of *Lactobacillus* (*P* < 0.05), while PBC significantly increased *Ruminococcaceae _UCG-005* (*P* < 0.05) (Fig. 4D).

**Fig. 5:**
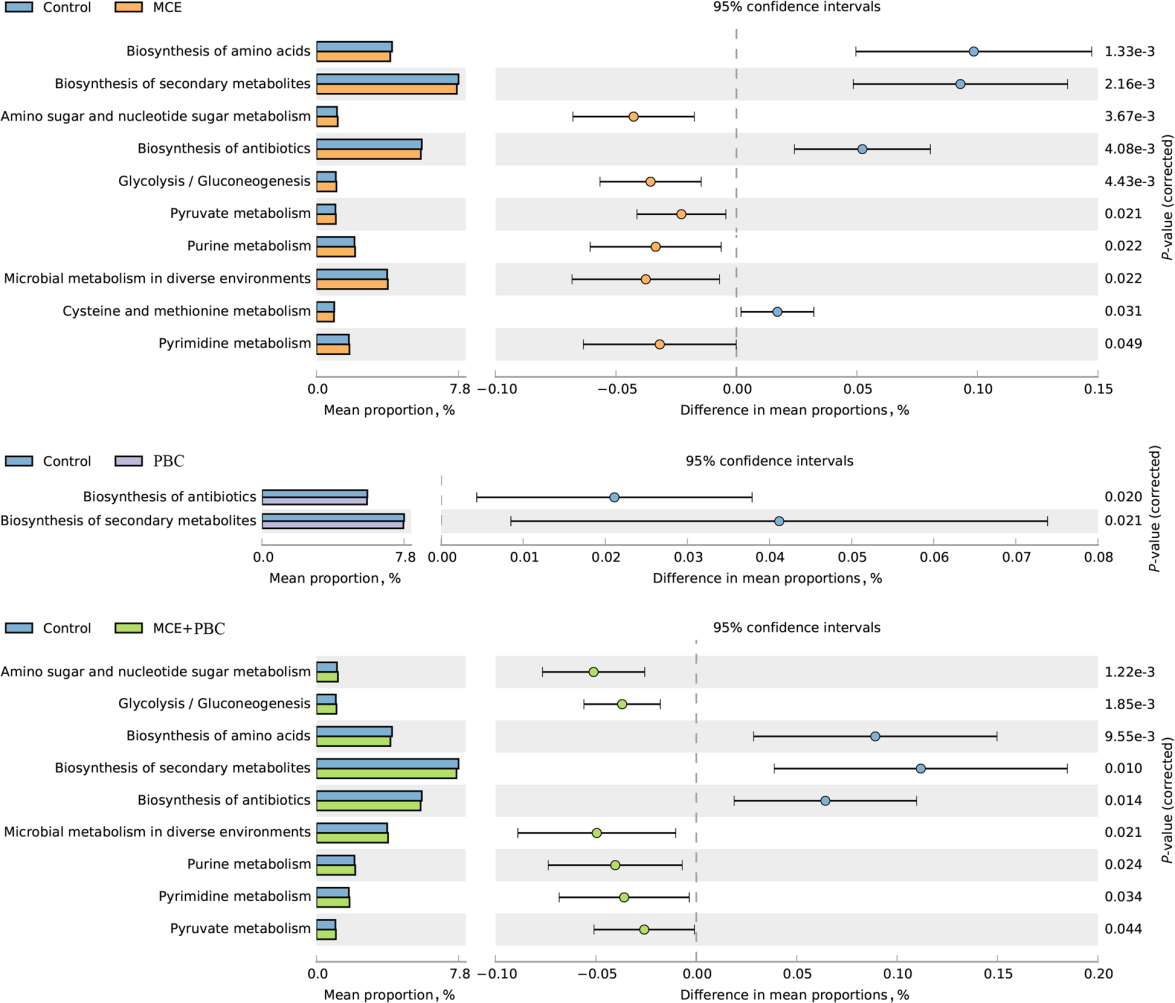
Comparison of predicted metabolic pathway abundances between the groups by STAMP. (**A**) Control vs. MCE; (**B**) Control vs. PBC; (**C**) Control vs. MCE + PBC. Confidence Interval was set at 95%

Phylogenetic investigation of communities by reconstruction of unobserved states (PICRUSt2) was further used to obtain the predicted metabolic functions of intestinal microbiota. The statistical analysis of taxonomic and functional profiles (STAMP) analysis based on level 3 of the microbial-predicted pathway functions further verified the differences in metabolic functions (Fig. 5). MCE and MCE + PBC treatment both significantly (*P* < 0.05) increased 6 metobolism pathways (amino sugar and nucleotide sugar metabolism, Glycolysis/Gluconeogenesis, microbial metabolism in diverse environments, purine metabolism, pyrimidine metabolism and pyruvate metabolism), whereas they decreased the biosynthesis of amino acids, biosynthesis of secondary metabolites and biosynthesis of antibiotics. Additionally, the pathway of the cysteine and methionine metabolism significantly decreased in the MCE group, and pathways associated with the biosynthesis of antibiotics and biosynthesis of secondary metabolites were also less active in the PBC group (*P* < 0.05).

### Concentrations of SCFAs in the cecal contents

**Fig. 6:**
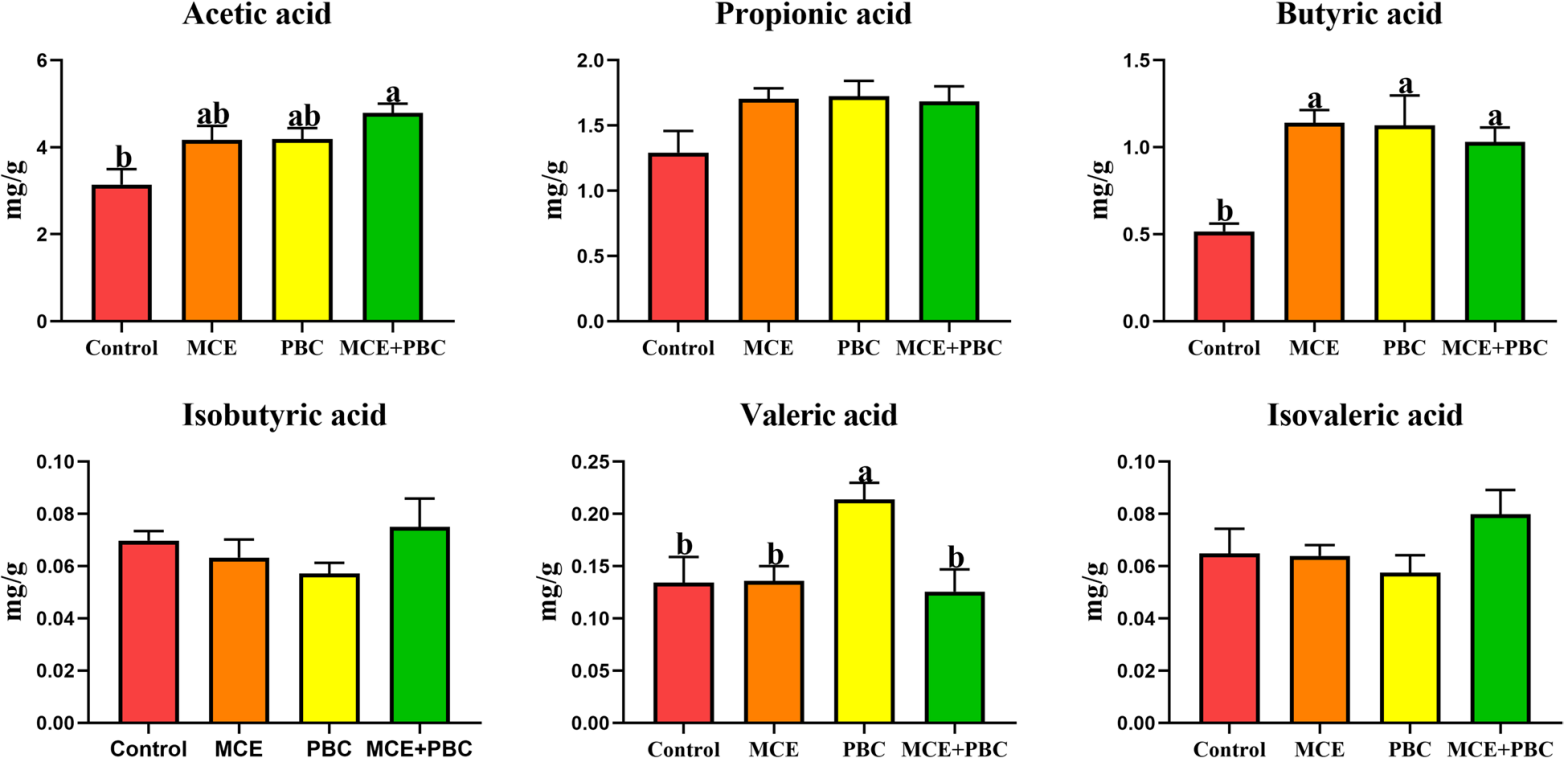
The concentrations of short-chain fatty acid (SCFAs) in the cecal contents of the laying hens. Data are presented as means ± SEM (*n* = 6). Different lowercase letters indicate a significant difference (*P* < 0.05)

The change of the intestinal microbiota will lead to change its metabolites, including short-chain fatty acids (SCFAs). Compared with the control group, the butyric acid level in the three treatment groups, the acetic acid level only in the MCE + PBC group, and the propionic acid only in the PBC group was significantly increased (*P* < 0.05) (Fig. 6). However, there were no significantly different in propionic acid, isobutyric acid and isovaleric acid among all groups.

### Intestinal physical barrier function

**Fig. 7:**
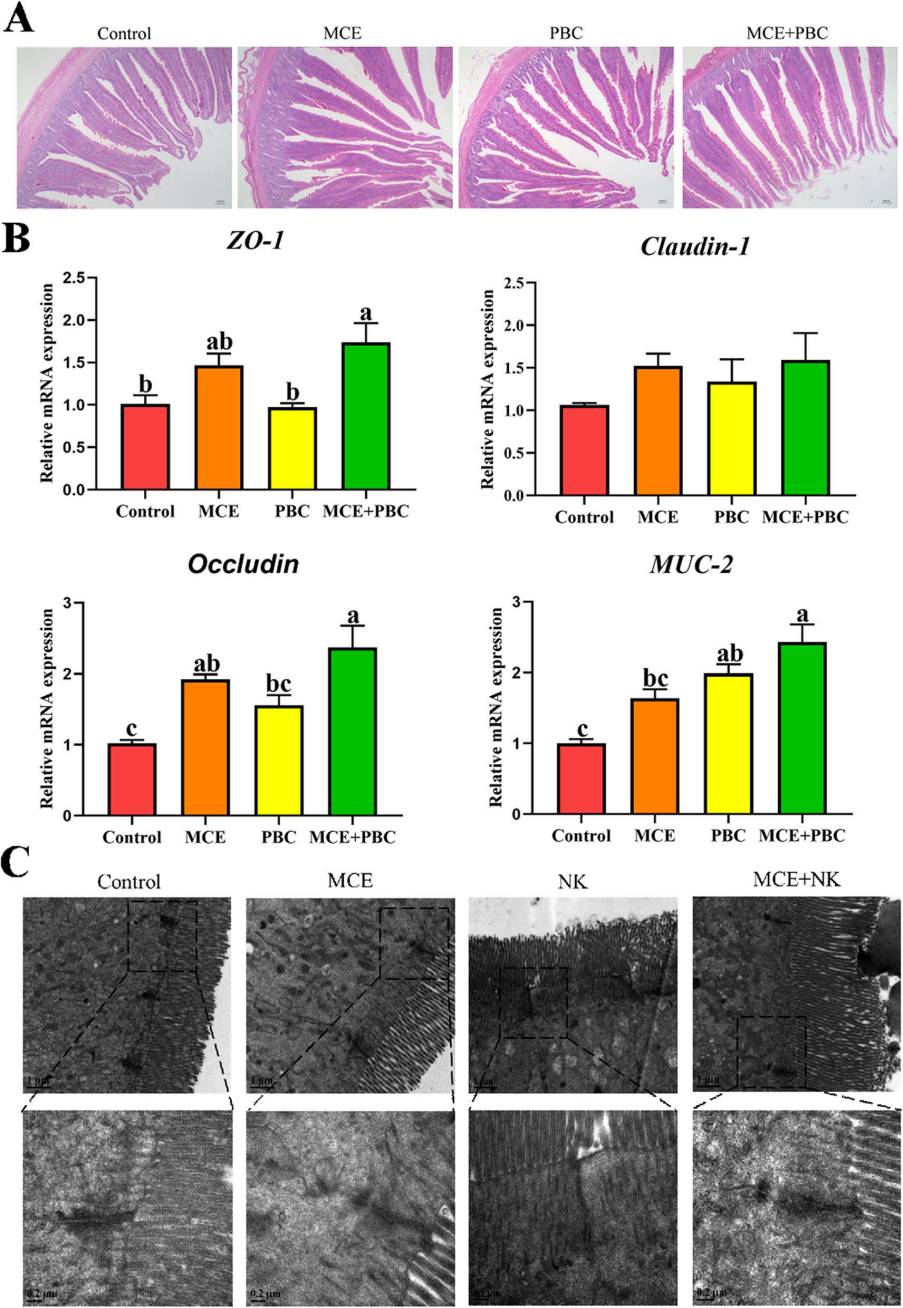
Effects of dietary supplementation with different biological feed additives on the Intestinal physical barrier function. (**A**) Histomorphology of the jejunum in laying hens. (**B**) The relative mRNA expressions of *ZO-1*, *Claudin-1*, *Occludin* and *MUC-2* were analyzed by real-time qPCR. Data are presented as means ± SEM (*n* = 3). Different lowercase letters indicate a significant difference (*P* < 0.05). *ZO-1*: zonula occluden 1; *MUC-2*: mucin-2. (**C**) Transmission electron micrographs of the jejunum microvilli in laying hens

Intestinal microbiota and its metabolites are closely related to intestinal physical barrier function**.** As shown in Table 4 dietary supplementation with MCE and PBC in combination significantly decreased the activities of LDH compared with the control and PBC groups (*P* < 0.05). The activities of MPO significantly decreased in the MCE group compared with the control group and in the MCE + PBC group compared with the PBC group (*P* < 0.05). The observation of the anatomic slices by light microscope showed that the villi of the jejunum in the MCE and PBC separately or mixed groups were more closely arranged and longer compared with the control group, and the MCE + PBC group was better than the MCE or PBC groups (Fig. 7A). The results of the mRNA expression of tight junction protein in jejunum was found that *Occludin* in the MCE group, *MUC-2* in the PBC group and *ZO-1*, *Occludin* and *MUC-2* in the MCE + PBC group were significantly increased (*P* < 0.05) (Fig. 7B). To further verify the above results, TEM detection showed that microvilli were ordered in all groups without significant difference, but the tight junctions (TJs) as well as adherent junctions (AJs) were longer, and desmosome (DS) was deeper in the MCE + PBC group compared with the other three groups (Fig. 7C). These data suggest that MCE or PBC can improve intestinal barrier function in laying hens, and the effect of MCE and PBC in combination is better.

**Table 4:** Serum biochemical parameters of intestinal function of laying hens^1^

Item	Control	MCE	PBC	MCE + PBC	SEM	*P*-value
LDH, U/L	7379.05^a^	6845.50^ab^	7172.01^a^	6448.17^b^	202.85	0.002
MPO, U/L	14.50^ab^	10.41^c^	16.63^a^	12.46^bc^	1.17	< 0.001

^1^Results are the means of each group of 6 laying hens. *LDH* lactic dehydrogenase *MPO* myeloperoxidase, *SEM* standard error of mean

^a^^−^^c^Value differences in the same row differ significantly (*P* < 0.05)

### Immune response and apoptosis

**Fig. 8:**
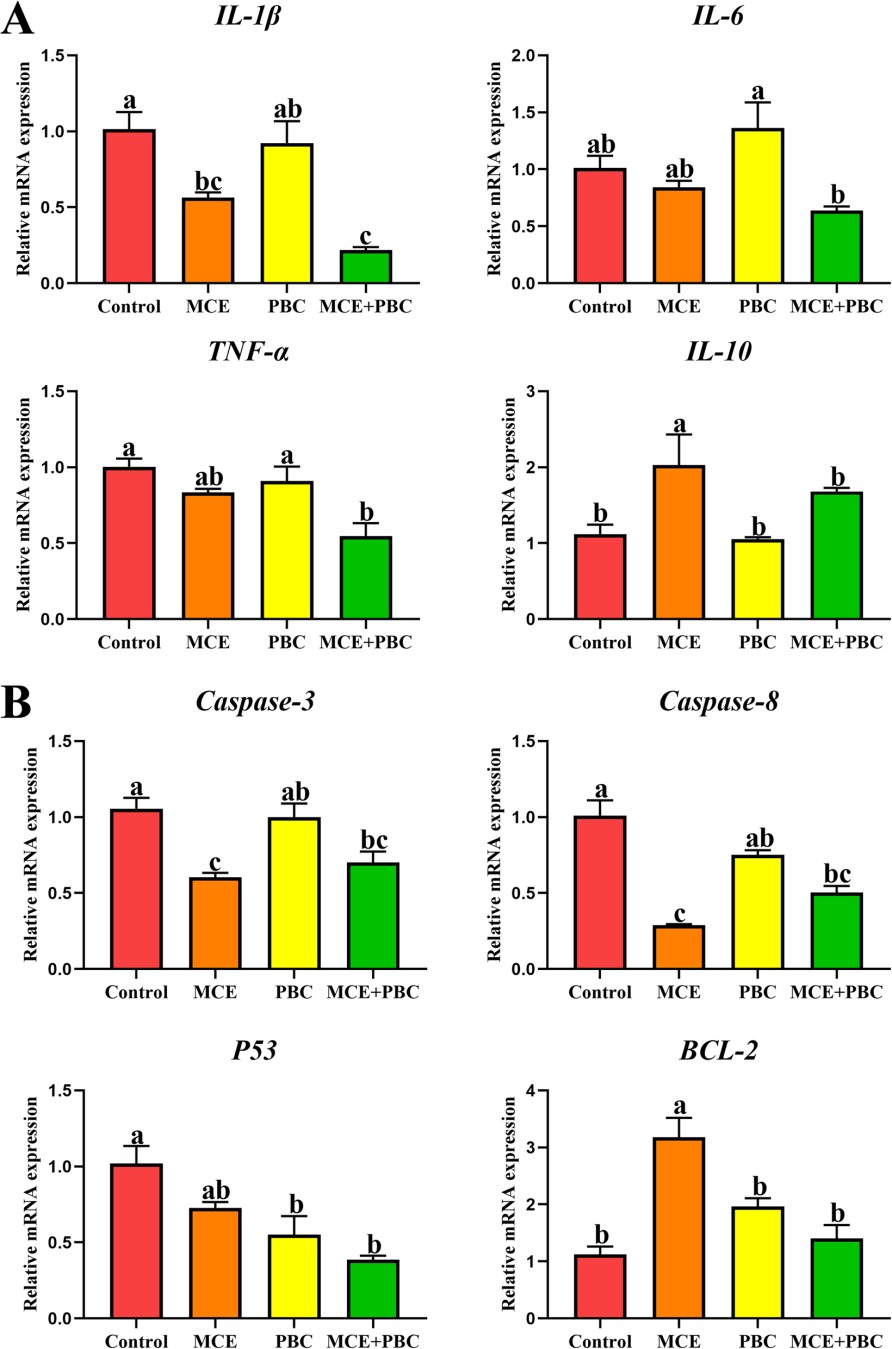
Effects of dietary supplementation with different biological feed additives on the relative mRNA expression of cytokines (**A**) and apoptosis factors (**B**). Data are presented as means ± SEM (*n* = 3). Different lowercase letters indicate a significant difference (*P* < 0.05). *IL*: interleukin; *TNF-α*: tumor necrosis factor-α

Intestinal microbiota and its metabolites affect not only physical barrier function but also immune function. The results of mRNA expression of cytokines in jejunum showed that compared with the control group, *IL*-1β in the MCE group and *IL*-1β and *TNF-α* in the MCE + PBC group were significantly decreased (*P* < 0.05), however, *IL*-10 in the MCE group was significantly up-regulated (*P* < 0.05), and there was no significant effect on the other cytokines (*P* > 0.05) (Fig. 8A). For apoptosis factors, compared with the control group, *Caspase 3* and *Caspase 8* in the MCE group, *P53* in the PBC group and *Caspase 3*, *Caspase 8* and *P53* in the MCE + PBC group were significantly decreased (*P* < 0.05), besides, *BCL-2* was significantly up-regulated in the MCE group (*P* < 0.05) (Fig. 8B). These results indicate that MCE and PBC separately or combined can enhance the immunity of laying hens, and the effect of MCE and PBC combined is better.

### Antioxidant status

For antioxidant status in the serum, compared to the control group, the T-AOC level in the MCE and in the MCE + PBC group were increased significantly (*P* < 0.05). The CAT activity in the MCE + PBC group was significantly higher than other three groups (*P* < 0.05). Activities of T-SOD and GSH-Px along with the concentrations of MDA were not affected among all groups. No significant differences for all antioxidant parameters were observed between the PBC group and control groups (*P* > 0.05) (Table 5).

**Table 5:** Antioxidant status of laying hens^1^

Item	Control	MCE	PBC	MCE + PBC	SEM	*P*-value
T-AOC, U/mL	5.06^b^	6.53^a^	5.83^ab^	6.73^a^	0.42	0.011
T-SOD, U/mL	106.02	109.48	104.71	111.30	4.28	0.679
CAT, U/mL	2.18^b^	2.62^b^	2.88^b^	5.81^a^	0.66	< 0.001
GSH-Px, U/mL	2287.61	2452.89	2667.77	2547.11	103.69	0.054
MDA, nmol/mL	2.73	2.25	2.56	2.15	0.25	0.333

^1^Results are the means of each group of 6 laying hens. *T-AOC* total anti-oxidant capacity, *T-SOD* total superoxide dismutase, *CAT* catalase, *GSH-Px* glutathione peroxidase, *MDA* malondialdehyde, *SEM* standard error of mean

^a^^−^^b^Value differences in the same row differ significantly (*P* < 0.05)

### Serum biochemical parameters

Gut health determines the health of the body health. The PBC and MCE + PBC groups significantly increased the concentration of TP (*P* < 0.05). No statistically significant differences were found in the other selected biochemical parameters among the control and different biological feed additives groups (*P* > 0.05) (Table 6). In addition, there was no significant effect on liver and kidney organ indexes (*P* > 0.05) (Supplementary Table S2).

**Table 6:** Serum biochemical parameters of liver and kidney function^1^

Item	Control	MCE	PBC	MCE + PBC	SEM	*P*-value
Liver function						
ALT, U/L	41.21	41.48	39.95	36.74	1.96	0.158
AST, U/L	44.02	41.28	43.23	43.24	1.49	0.635
AKP, U/L	165.27	193.80	153.77	183.84	14.82	0.223
TC, mmol/L	4.14	5.03	4.08	4.92	0.56	0.616
TP, g/L	51.48^b^	60.31^ab^	67.71^a^	67.81^a^	4.27	0.010
ALB, g/L	32.28	37.46	37.26	36.27	3.67	0.758
Kidney function						
BUN, mmol/L	2.96	3.05	2.79	2.94	0.11	0.527
UA, μmol/mL	7.58	6.69	6.47	7.18	0.38	0.158

^1^Results are the means of each group of 6 laying hens. *ALT* alanine aminotransferase, *AST* aspartate aminotransferase, *AKP* alkaline phosphatase, *TC* total cholesterol, *TP* total protein, *ALB* albumin, *BUN* blood urea nitrogen, *UA* uric acid, *SEM* standard error of mean

^a^^−^^c^Value differences in the same row differ significantly (*P* < 0.05)

### The correlations among antioxidant indicators, hormone and intestinal microbiota

**Fig. 9:**
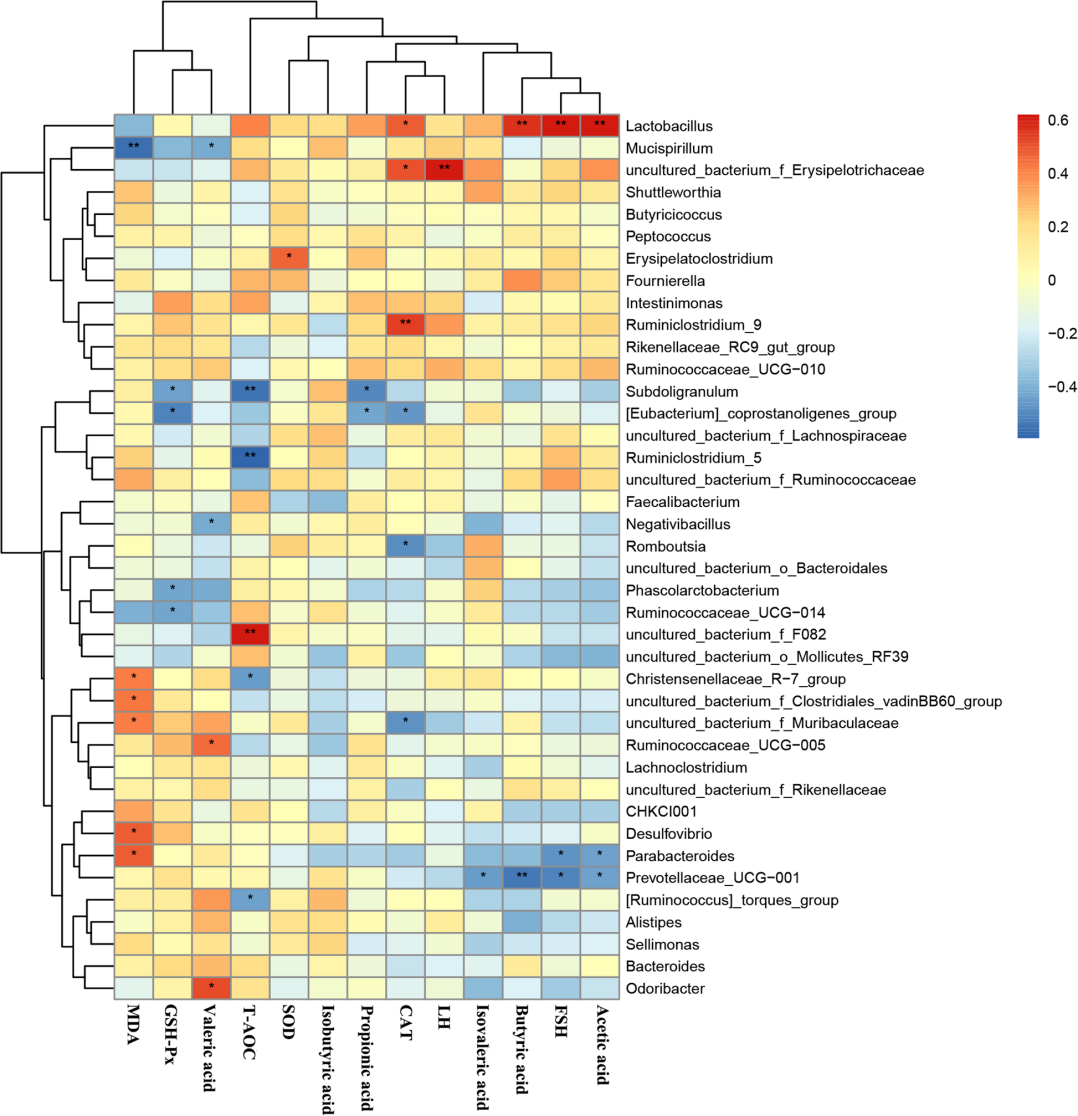
Heatmap of Spearman’s correlation between the Intestinal microbiota and antioxidant indicators or gonadotropin at the genus level. The intensity of the colors represented the degree of association (red, negative correlation; blue, positive correlation). Significant correlations were marked by *P < 0.05; **P < 0.01; ***P < 0.001

To further understand the role of whole intestinal microbiota in regulating the concentrations of SCFAs, gonadotropin and antioxidants by Spearman’s correlation analysis (Fig. 9). *Lactobacillus* had a positive correlation (*P* < 0.01) with levels of acetic acid and butyric acid and FSH, while *Parabacteroides* (*P* < 0.05) and *prevotellaceae_UCG-001* (*P* < 0.05) were negatively correlated with acetic acid and FSH. Moreover, *prevotellaceae_UCG-001* were negatively correlated with butyric acid (*P* < 0.01) and isovaleric acid (*P* < 0.05). The *uncultured_bacterium_f_F082* positively (*P* < 0.01) correlated with T-AOC, but was negatively correlated with *Ruminiclostridium_5* (*P* < 0.01), *Subdoligranulum* (*P* < 0.01) *[Ruminococcus]_torques_group* (*P* < 0.05) and *Christensenellaceae_R-7_group* (*P* < 0.05). Among the microbial genera up-regulated by MCE and PBC separately or in combination, *Lactobacillus* expects the previously mentioned that was positively correlated with FSH and the levels of acetic acid and butyric acid, which also was significantly positive correlation with CAT (*P* < 0.05). In addition, CAT also significantly correlated with *Ruminiclostridium_9* (*P* < 0.01) and *uncultured_bacterium_f_Erysipelotrichaceae* (*P* < 0.05), and negatively correlated with *[Eubacterium]_coprostanoligenes_group*, *Romboutsia*, and *uncultured_bacterium_f_Muribaculaceae* (*P* < 0.05).

## Discussion

Plant extracts and probiotics exert lots of effects, including improving the production performance of poultry, preventing disease infection, enhancing body health and so on [39, 40]. This study showed that MCE or PBC improved the LR and ADEM with no significant difference. Our results are similar to those of Guo et al. [18], who reported that dietary supplementation with MCE had a positive impact on laying performance, and Wang et al. [28] reported that average daily gain was increased and average daily feed intake and feed conversion ratio was decreased for broilers fed with a diet supplemented with BaSC06. Interestingly, that supplement with MCE and PBC in combination to the diet, significantly improved the performance of laying hens more than the control group. Previous studies have shown that TCM (*Taraxacum* extracts, *Astragalus* polysaccharides, and total flavonoids) and probiotics (*Lactobacillus* and *Bacillus*) separately or in combination had different degrees of protective effect, including increased BW gain, decreased the diarrhea rate and mortality of broilers. Among them, TCM and probiotics in combination had the best effect, and this may contribute to the synergistic or additive effect of this combination therapy [31]. In this study, the synergistic effect of MCE and PBC greatly improved the laying rate and average daily egg production of laying hens.

Reproductive organ development, follicular development, maturation, and ovulation in laying hens are mainly regulated by the hypothalamus–pituitary–gonad axis [41], and gonadotropins are essential for regulating ovarian development, steroid production, and gametogenesis. Follicle-stimulating hormone (FSH) promotes follicular development and luteinizing hormone (LH) regulates oocyte preovulation, ovulation, and luteal formation [42]. Therefore, there is a certain relationship between egg production and reproductive hormone content of laying hens. In this study, the combined use of MCE and PBC increased the levels of FSH and LH in serum, which can increase the number of follicles, stimulate follicle growth and maturation, which was confirmed by the egg production rate of the group mentioned above. The oocyte is surrounded by somatic cells (granulosa cells and theca cells), which make up the ovarian follicle. Among them, only somatic cells can express gonadotropin receptors, which FSH receptor and LH/chorionic gonadotropin receptor are expressed on the somatic cells [43]. The significant upregulation the mRNA expressions of *FSHR* and *LHR* in preovulation granulosa cells of laying hens fed with MCE and PBC combination may be related to the secretion of more hormones. Increased levels of *FSHR* and *LHR* can greatly increase follicular reactivity to FSH and LH, and ultimately promote follicular maturation [44]. In addition, *ERβ* in differentiating granulosa cells of the ovary, whereas *ERα* was predominantly seen in interstitial cells [45]. *ERβ* is the main estrogen receptor in ovary, and adult ovary is the site with the highest expression level of *ERβ* [46]. We monitored the mRNA expression of *ERβ* and found the same trend as *LHCGR* and *FSHR*. It is speculated that *ERβ* is likely to play a key role in regulating ovarian function, including gonadotropin-mediated function.

Intestinal microbiota plays an important role in maintaining gut homeostasis. Numerous studies have shown that both plant extracts and probiotics directly affect intestinal microbiota first. Intestinal microbiota can coevolve with the host to form a stable intestinal microbial environment and provide a wide range of biological functions for the host, which may vary from individual to individual due to genetic and environmental factors [47, 48]. In this study, there was no significant effect on α diversity in all treatment groups, but PCoA results clearly show that dietary supplementation of MCE and PBC separately or in combination can alter microbial community structure, which is partly consistent with previous studies [49, 50]. In the cecum of laying hens, Bacteroidetes were the most dominant phylum, followed by Firmicutes, which was consistent with the results of Guo et al. [49]. But interestingly, all treatments decreased the relative abundance of WPS-2, which includes bacteria with diverse metabolic capabilities [51]. In previous studies, MCE can significantly improve the relative abundance of *Lactobacillus* in the cecum of the laying hens [49], which is consistent with our results in this study, using of MCE or combined with PBC significantly increased the relative abundance of *Lactobacillus*.

As is well known, short-chain fatty acids (including acetate, propionate, butyrate and so on) are produced by the fermentation of undigested carbohydrates by intestinal microorganisms [52], which can promote the intestinal morphology and immune state of animals [53, 54], and can also be absorbed by the intestinal epithelial cells of animals as energy for growth and production [55]. Thus, the concentration of short-chain fatty acids in the cecum can be used as an indicator of gut health, indicating that the cecum is rich in beneficial bacteria [56]. Interestingly, in this experiment, the concentrations of butyric acid in the cecum were significantly increased in all three treatment groups compared to the control group, but only the MCE + PBC significantly increased the concentration of acetic acid.

Biochemical blood parameters usually reflect the health of animals, which are vital indicators of the nutritional and physiological status of birds and mammals [57]. The levels of lactate dehydrogenase (LDH) changed in the serum become a common marker of tissue damage and disease [58, 59] and maladjustment of myeloperoxidase (MPO) release can cause tissue damage [60]. In this study, we found the use of MCE and PBC in combination reduced the levels of LDH and MPO in the serum, indirectly indicating the improved intestinal integrity.

The intestinal epithelial integrity serves as a physical barrier against enteric pathogen invasion and is responsible for nutrient absorption and waste secretion [61], which is mainly composed of intestinal epithelial cells and junctional complexes such as tight junctions and gap junctions [62–64]. Moreover, mucus layers are constructed of goblet cell-secreted MUC-2 protein, which can prevent the invasion of pathogenic bacteria [65]. Our study showed that the villi of the jejunum in the MCE and PBC separately or mixed groups were more closely arranged and longer compared with the control group, and the morphology of the MCE + PBC group was better than the MCE or PBC groups. Further to explore the intrinsic mechanism, we found the mRNA expressions of *ZO-1*, *Occludin* and *MUC-2* were significantly increased in the MCE + PBC group. Similarly, Liu et al. [16] found that boosts volumes of *ZO-1* and *Claudin-1* proteins greatly by being treated with MCE. Meanwhile, *Bacillus* improved intestinal mucosa structure and promoted the mRNA expression of the tight junction protein occluding [66, 67]. Next, the results of the TEM to further confirm the above mentioned, which the TJs as well as AJs were longer, and DS was deeper in the MCE + PBC group. These results above indicate that dietary supplementation with the MCE and PBC in combination is most beneficial for the development of intestinal mucosa and enhancement of the intestinal epithelial integrity of laying hens.

Homeostasis of intestinal physiology depends on complex communication and regulation between immune cells, cytokines, intestinal microbiota and host [68, 69]. If this homeostasis is caused by an environmental injury (infectious or non-infectious), it activates a highly regulated cascade of physiological and immune events leading to an inflammatory response [70]. *TNF-α* and *IL-1β* are important pro-inflammatory cytokines that regulate host immunity to a variety of pathogens through immune cell differentiation, proliferation and apoptosis [71]. Due to the long period of laying eggs, disruption of tight connections and dysregulation of the microbiome can lead to inflammation and tissue damage [72]. In this study, inhibition of *IL-1β* and *TNF-α* by the dietary supplementation of MCE and PBC reduced inflammation and improved intestinal health, as demonstrated by the increased tight junction protein and the improved intestinal barrier. In fact, previous studies have shown that the anti-inflammatory effects of MCE have been well demonstrated in pharmacological experiments by inhibiting pro-inflammatory cytokines and reducing inflammatory cell recruitment, thereby reducing tissue damage [6]. The significant down-regulation mRNA expressions of *Caspase 3* and *Caspase 8*, which are related to apoptosis [73], prove that MCE has good anti-inflammatory and anti-apoptotic properties, and the effect is better than when combined with PBC. However, the exact mechanism needs further investigation.

Oxidative stress occurs due to the production of reactive oxygen species (ROS) due to increased metabolic activity during egg production [74], which is the imbalance between the production and clearance of ROS, whose level exceeds the endogenous protective mechanism of the body [75]. Excessive ROS attacks cell components such as lipids, protein and DNA, resulting in lipid peroxidation of cell membrane, mitochondrial dysfunction and DNA breakage, and ultimately terminated by cell death [76]. Total antioxidant capacity reflects the scavenging capacity of the antioxidant system to oxygen free radicals. There is a close relationship between the antioxidant capacity of the body defense system and the health degree. Superoxide dismutase can clear superoxide anion radical (O_2_^−^·) disproportionation to generate oxygen and hydrogen peroxide, then catalase can promote the decomposition of H_2_O_2_ into molecular oxygenated water to remove hydrogen peroxide in the body, thus protecting cells from damage [77]. In this study, we found that the use of MCE and PBC in combination had a better effect on antioxidants than the separate use, especially in the activity of catalase. And the results of this study are in agreement with the relevant study, which combined the use of probiotic strains of bacteria and *Quercus cortex* extract helped to increase the antioxidant activity of the organism [33]. In addition, there are research reports that egg production was negatively correlated with oxidative stress [78], but animals can boost antioxidant capacity by maintaining the integrity of the gut barrier [79].

*Lactobacillus* is involved in the synthesis of some essential vitamins and organic acids, which contributes significantly to nutrient absorption and related intestinal function [80]. Moreover, the increase of the relative abundance of *Lactobacillus* may compete against harmful bacteria and play a positive role in the intestinal health of laying hens, and *Lactobacillus* can have a positive effect on FSH, acetic acid, butyric acid as well as CAT and a negative effect on MDA in the function prediction of this experiment. In addition, the metagenome prediction results showed that the use of MCE separately or combination with PBC all could significantly increase the carbohydrate metabolism-related pathways and reduce the nucleotides biosynthesis-related pathways in intestinal microorganisms, which can promote production performance and weaken the growth of microbes.

## Conclusion

**Fig. 10:**
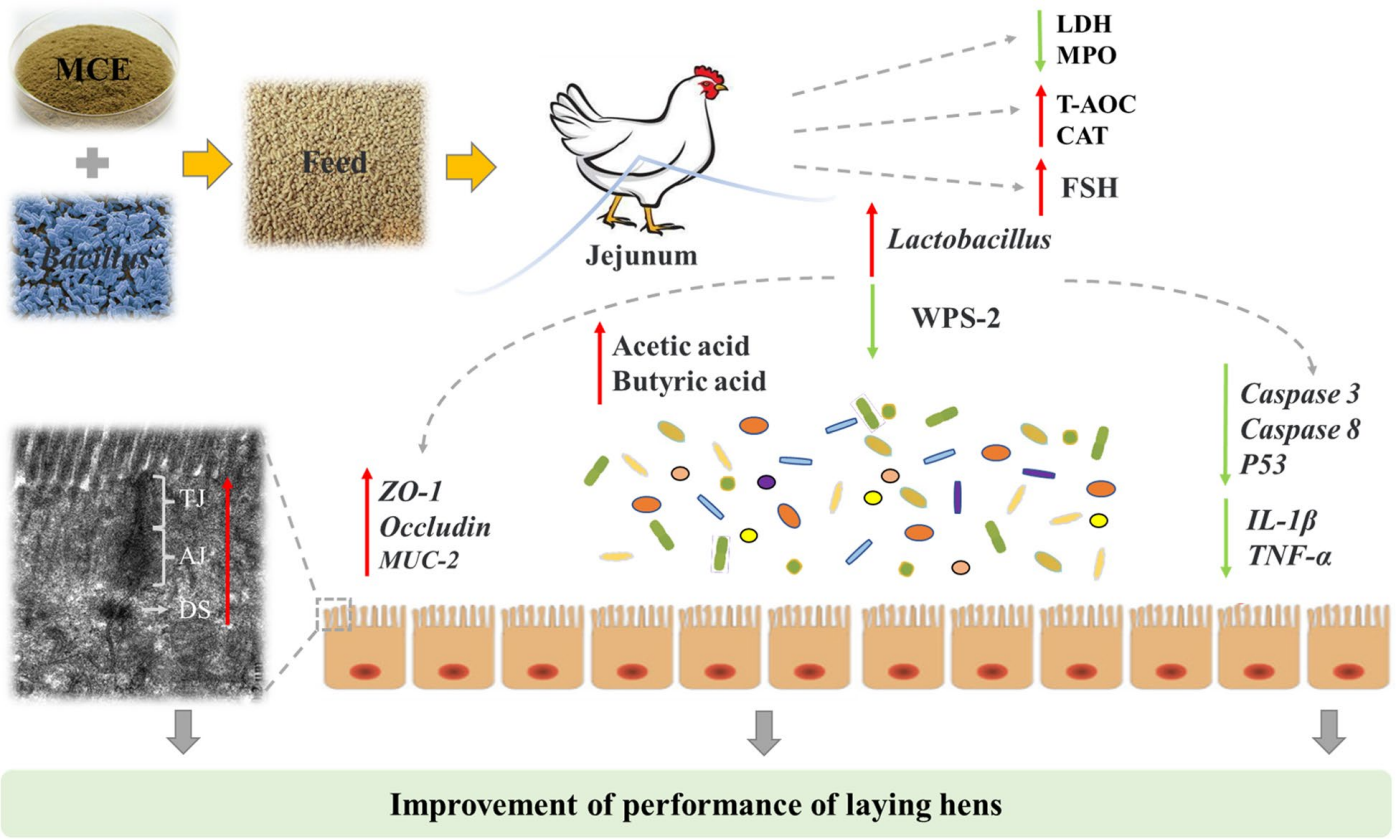
Graphical summary of the effect of *Macleaya cordata* extract and *Bacillus* in combination on the performance and intestinal health of laying hens

In conclusion, this study shows that dietary supplementation of MCE and *Bacillus* in combination increases laying rate and average daily egg production of laying hens by improving antioxidant capacity, intestinal morphology, epithelial barrier function, immune status, the content of gonadotropin and the concentrations of short-chain fatty acids in the cecum, which could be related to the regulation of intestinal microbiota (Fig. 10). These findings contribute to a deeper understanding of the potential mechanisms by which combined the use of MCE and *Bacillus* may improve production performance and intestinal health in laying hens.

## Supplementary information

**Additional file 1:** **Table S1.** Sequences of oligonucleotide primers used for RT-qPCR. **Table S2.** Organs indices of laying hens fed different biological feed additives^1^.

## Data Availability

The datasets produced and/or analyzed during the current study are available from the corresponding author on reasonable request.

## Abbreviations

MCE: *Macleaya cordata* extract
LR: Laying rate
ADEM: Average daily egg mass
ADFI: Average daily feed intake
FCR: Feed conversion ratio
FSH: Follicle-stimulating hormone
LH: Luteinizing hormone
ER: Estrogen receptor
FSHR: Follicle-stimulating hormone receptor
LHCGR: Luteinizing hormone/choriogonadotropin receptor
LDH: Lactic dehydrogenase
MPO: Myeloperoxidase
ZO-1: Zonula occluden 1
MUC-2: Mucin-2
IL: Interleukin
TNF-α: Tumor necrosis factor-α
T-AOC: Total anti-oxidant capacity
T-SOD: Total superoxide dismutase
CAT: Catalase
GSH-Px: Glutathione peroxidase
MDA: Malondialdehyde
ALT: Alanine aminotransferase
AST: Aspartate aminotransferase
AKP: Alkaline phosphatase
TC: Total cholesterol
TP: Total protein
ALB: Albumin
BUN: Blood urea nitrogen
UA: Uric acid
