# Supplementary material for: Dietary supplementation of Macleaya cordata extract and Bacillus in combination improve laying performance by regulating reproductive hormones, intestinal microbiota and barrier function of laying hens
Source: J Anim Sci Biotechnol. 2022 Oct 13;13:118. doi: 10.1186/s40104-022-00766-4 (PMC9559840; doi:10.1186/s40104-022-00766-4)
Supplement: Supplementary file 1 — Additional file 1: Table S1. Sequences of oligonucleotide primers used for RT-qPCR. Table S2. Organs indices of laying hens fed different biological feed additives1. [file 40104_2022_766_MOESM1_ESM.doc]

**Supplementary Materials**

**Table S1 Sequences of oligonucleotide primers used for RT-qPCR**

| Gene | Primer Sequence (5’ to 3’) | Product size, bp | Annealing temperature, °C | Accession No. |
| --- | --- | --- | --- | --- |
| ZO-1 | F: GCTCACAAGCTACGCAAAAA  R: ACCATCTGCCTTTCCTTCAG | 150 | 57 | XM_015278981.1 |
| Claudin-1 | F: TATGGCAACAGAGTGGCTCG  R: TCAGGACAGCGGCATTGTAG | 294 | 60 | NM_001013611.2 |
| Occludin | F: TCTGCCTCATCTGCTTCTTC  R: TTCTTCACCCACTCCTCCA | 127 | 58 | NM_205128.1 |
| MUC-2 | F: GCCTGCCCAGGAAATCAAG  R: CGACAAGTTTGCTGGCACAT | 59 | 59 | XM_040673077.1 |
| BCL-2 | F: TCTTCCGTGATGGGGTCAAC  R: AAGGCATCCCATCCTCCGTT | 184 | 60 | NM_205339.2 |
| P53 | F: CGCCGTGGCCGTCTATAAG  R: GTACAGTCAGAGCCCACCTCG | 219 | 63 | NM_205264.1 |
| Caspase-3 | F: ACTCTGGAAATTCTGCCTGATGACA  R: CATCTGCATCCGTGCCTGA | 130 | 60 | NM_204725.2 |
| Caspase-8 | F: CATTGCATGGGCTGCTTAAA  R: CACCTCCCCTACGCCTATCT | 149 | 59 | NM_204592.4 |
| IL-1β | F: CGACATCAACCAGAAGTGCTT  R: GTCCAGGCGGTAGAAGATGA | 298 | 59 | NM_204524.2 |
| IL-6 | F: CTCCTCGCCAATCTGAAGTC  R: CCTCACGGTCTTCTCCATAAAC | 99 | 60 | NM_204628.2 |
| TNF-α | F: GGACAGCCTATGCCAACAAG  R: GCGGTCATAGAACAGCACTAC | 81 | 60 | NM_204267.2 |
| IL-10 | F: TGCTGGATGAGTTTAAGGGGAC  R: CCCATGCTCTGCTGATGACT | 121 | 60 | NM_001004414.2 |
| ER-α | F: TGGGCAAAGAGAGTTCCAGG  R: ACATTTCCCTTGATTCCTGTCCA | 174 | 59 | NM_205183.2 |
| ER-β | F: GGCTGCAACCCGTGTAAAAG  R: GCCCAGCCAATCATGTGAAC | 189 | 60 | NM_204794.3 |
| FSHR | F: ACGTGTTCCATCTCAGCTTTT  R: TTGGTGAGGACAAATCTCAGTTC | 293 | 57 | NM_205079.2 |
| LHCGR | F: CGAATCGCTGACACTCAAACT  R: CTCTCAGGGCATCGTTGTGT | 137 | 59 | NM_204936.2 |
| β-actin | F: TATGTGCAAGGCCGGTTTC  R: TGTCTTTCTGGCCCATACCAA | 110 | 58 | NM_205518.2 |

F: forward primer; R: reverse primer. *ZO-1*: zonula occludens-1; *MUC-2*: mucin-2; *IL*: interleukin; *TNF-α*: tumor necrosis factor-α; *ER*: estrogen receptor; *FSHR*: follicle-stimulating hormone receptor; *LHCGR*: luteinizing hormone/choriogonadotropin receptor.

**Table S2 Organs indices of laying hens fed different biological feed additives^1^**

| Item | Control | MCE | PBC | MCE+PBC | SEM | *P*-value |
| --- | --- | --- | --- | --- | --- | --- |
| Liver index, g/kg | 22.33 | 23.29 | 21.33 | 22.32 | 0.59 | 0.134 |
| Spleen index, g/kg | 0.97 | 0.93 | 0.99 | 0.97 | 0.03 | 0.532 |

^1^ Results are the means of each group of 6 laying hens. In the same row, *P* > 0.05 indicates no significant difference of four groups. SEM: standard error of mean
